# Epigenetics of Hepatic Insulin Resistance

**DOI:** 10.3389/fendo.2021.681356

**Published:** 2021-05-11

**Authors:** Hannah Maude, Claudia Sanchez-Cabanillas, Inês Cebola

**Affiliations:** Section of Genetics and Genomics, Department of Metabolism, Digestion and Reproduction, Imperial College London, London, United Kingdom

**Keywords:** epigenetics (DNA methylation, histone modifications), liver, insulin resistance, type 2 diabetes, NAFLD (non-alcoholic fatty liver disease)

## Abstract

Insulin resistance (IR) is largely recognized as a unifying feature that underlies metabolic dysfunction. Both lifestyle and genetic factors contribute to IR. Work from recent years has demonstrated that the epigenome may constitute an interface where different signals may converge to promote IR gene expression programs. Here, we review the current knowledge of the role of epigenetics in hepatic IR, focusing on the roles of DNA methylation and histone post-translational modifications. We discuss the broad epigenetic changes observed in the insulin resistant liver and its associated pathophysiological states and leverage on the wealth of ‘omics’ studies performed to discuss efforts in pinpointing specific loci that are disrupted by these changes. We envision that future studies, with increased genomic resolution and larger cohorts, will further the identification of biomarkers of early onset hepatic IR and assist the development of targeted interventions. Furthermore, there is growing evidence to suggest that persistent epigenetic marks may be acquired over prolonged exposure to disease or deleterious exposures, highlighting the need for preventative medicine and long-term lifestyle adjustments to avoid irreversible or long-term alterations in gene expression.

## Introduction

The liver has a pivotal role in metabolic homeostasis. As an endocrine organ, it is part of a highly interconnected network that can influence whole-body metabolic health. After consuming food, the liver switches from producing glucose *via* glycogenolysis (conversion of glycogen stores) and gluconeogenesis (*de novo* glucose production), to taking up and storing glucose from the blood. This switch is stimulated by the anabolic hormone, insulin, which is released by pancreatic β-cells in response to high blood glucose levels following a meal. Insulin is required to keep blood glucose levels within a tightly controlled range. In several pathological states, however, peripheral tissues can lose their responsiveness to insulin, a state commonly known as insulin resistance (IR).

IR is a common denominator of multiple metabolic deficiencies, including elevated fasting glucose, elevated triglycerides, reduced high-density lipoprotein cholesterol and hypertension (1). Continued IR can eventually lead to disease, including type 2 diabetes (T2D) and non-alcoholic fatty liver disease (NAFLD), which are estimated to affect 9.3% and 25% of the global population, respectively (2, 3). IR is also a major risk factor for cardiovascular disease (4) and cognitive decline in age-related degenerative diseases, such as Alzheimer and Parkinson’s disease (5), while also being a defining feature of gestational diabetes mellitus (6) and polycystic ovary syndrome (7). It is therefore of concern that estimates of IR prevalence in children range between 3-44% (8), presenting the picture of a significant public health issue. The association of IR with multiple leading causes of global morbidity and mortality motivates this review to discuss the progress in understanding its underlying biology.

The centrality of the hepatic insulin response in cardiometabolic homeostasis was elegantly demonstrated by Ronald C. Kahn’s team over two decades ago, who observed that mice with liver-specific knockout of the insulin receptor exhibited hyperinsulinemia, abnormal levels of lipids in the blood (dyslipidaemia) and a proatherogenic lipoprotein profile (9, 10). Moreover, hepatic IR can contribute to a destructive cycle by inducing hyperplasia of pancreatic β-cells and hyperinsulinemia (11) and may precede the onset of whole-body IR and T2D (12, 13). Under physiological conditions, insulin inhibits hepatic glucose production and increases fatty acid and triglyceride biosynthesis. Paradoxically, hepatic IR results in an increase of both glucose and lipid production, a state referred to as ‘selective hepatic insulin resistance’ (14). Consequently, hepatic IR causes both hyperglycaemia and hypertriglyceridemia, resulting in a tight association with NAFLD, a spectrum of disease states characterized by excessive accumulation of fat in the liver (hepatic steatosis).

IR is a multifactorial trait, being influenced by both environmental and lifestyle risk factors, as well as inherited genetic variation. While genetic variation refers specifically to changes in the DNA code, epigenetics considers additional modifications to the genome which can be transmitted to daughter cells independently of changes in the DNA sequence. An expanding repertoire of research is revealing a central role of epigenetics in diverse aspects of disease biology, including as long-term manifestations of environmental exposures and effectors of underlying pathological mechanisms. The epigenome also offers potentially transformative disease biomarkers and therapeutic targets. As such, this review will provide an overview of the current evidence detailing the roles of epigenetic and gene regulatory mechanisms of hepatic IR.

## The Liver Epigenome

**Figure 1:**
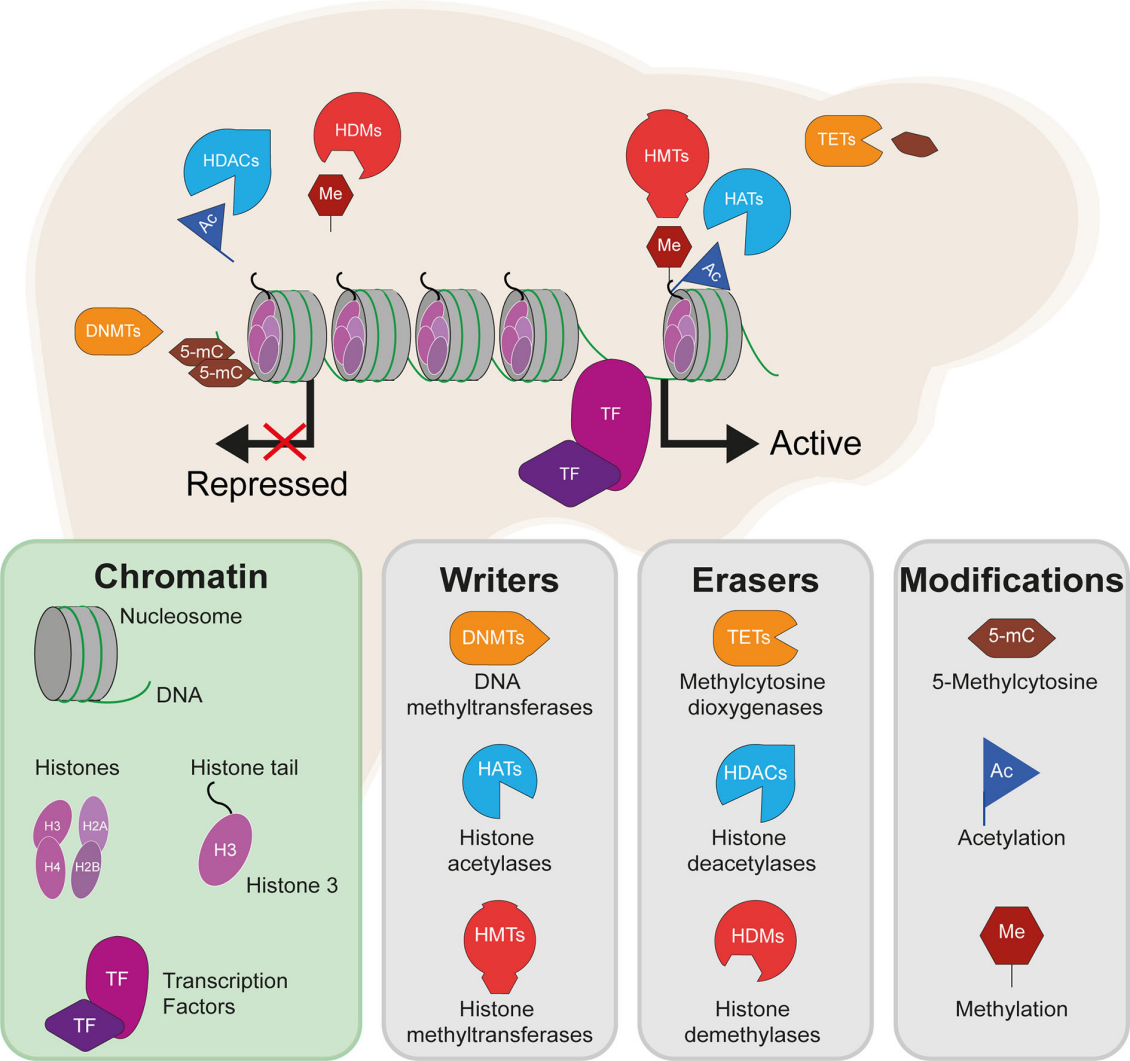
DNA methylation, histone methylation and acetylation and their epigenetic machinery: sources of gene regulation in the liver. Repressed gene expression is shown where epigenetic modifiers remove active histone marks (e.g. H3K27ac and methylation of H3K4), including histone deacetylases (HDACs) and histone demethylases (HDMs). Active gene expression is shown where active histone marks are deposited by histone acetyltransferases (HATs) and histone methyltransferases (HMTs). DNA methylation is here shown to repress gene expression, which typically occurs following the methylation of gene promoters and is deposited by DNA methyltransferases (DNMTs) and removed by methylcytosine dioxygenases (TET enzymes).

The liver epigenome is largely disrupted across IR-related disease states. While there are a variety of potential epigenetic modifications, those which feature most in the literature and in this review include DNA methylation, deposited by DNA methyltransferases (DNMTs) and removed by T5-methylcytosine hydroxylase (TET) enzymes; histone acetylation, deposited by histone acetyltransferases (HATs) and removed by histone deacetylases (HDACs); and histone methylation, deposited by histone methyltransferases (HMTs) and removed by histone demethylases (HDMs) (**Figure 1**). These modifications include both permanent marks, which contribute to cell identity and metabolic zonation in the liver^1^, and dynamic marks, which regulate gene expression in response to various stimuli. By way of example, environmental exposures can be seen in the accumulating differences in the epigenomes of identical twins over time (16). As a result, the disruption of the hepatic epigenome across IR-related disease states stretches from the manifestation of long-term (e.g. *in utero*) to short-term (e.g. dietary) exposures.

DNA and histone modifications alter gene expression levels by adding and removing chemical groups to DNA and the histone proteins around which the DNA is wrapped. Such modifications may alter how tightly the DNA is packaged and how accessible it is to transcription factors (TFs), for example. More broadly, these can affect the so-called *regulatory landscape* of genes, for example by changing the activity of *cis*-regulatory elements such as transcriptional enhancers which may reach physical proximity with target gene promoters *via* DNA looping (17). The liver epigenome also hosts a broad array of noncoding RNAs that can influence gene expression programmes. However, the role of noncoding RNAs in hepatic IR are beyond the scope of this review and the reader is directed to other recent reviews that cover this topic in detail (18, 19).

The functions of specific epigenetic modifications depend on their context, including their position relative to a gene body or the specific amino acid modified. DNA methylation, which most commonly occurs in mammals at the C5 position of cytosine bases at CpG dinucleotides, is known to be a particularly stable and long-term mark which is typically associated with transcriptional repression, although studies have revealed context-dependent functions in both genome stability and gene transcription (20). Changes in DNA methylation, particularly at promoter ‘CpG islands’ which are characterised by dense regions of largely unmethylated CpGs, can have a significant impact on gene expression levels (20). The liver has zonated patterns of DNA methylation and associated gene expression across the pericentral, intermediate and periportal liver, of which an interesting example is the differential methylation of HNF4α (the core hepatic TF, hepatocyte nuclear factor 4α) recognition sites, which mediate its zone-dependent effects and regulation of target genes (21). Of the histone marks, histone acetylation is generally associated with increased DNA accessibility and increased gene expression, while histone methylation can both activate and repress gene expression. For example, H3K9me2 occurs at repressive heterochromatin and H3K4me1 and H3K4me3 are found at enhancers and promoters, respectively. The application of next-generation sequencing to characterise genomic regions that are enriched for these modifications has truly transformed the epigenomics^2^ field and has enabled the characterization of a regulatory code, whereby specific combinations of epigenetic marks associate with specific types of DNA element, such as H3K4me1 and H3K27ac at active transcriptional enhancer elements (22).

**Figure 2:**
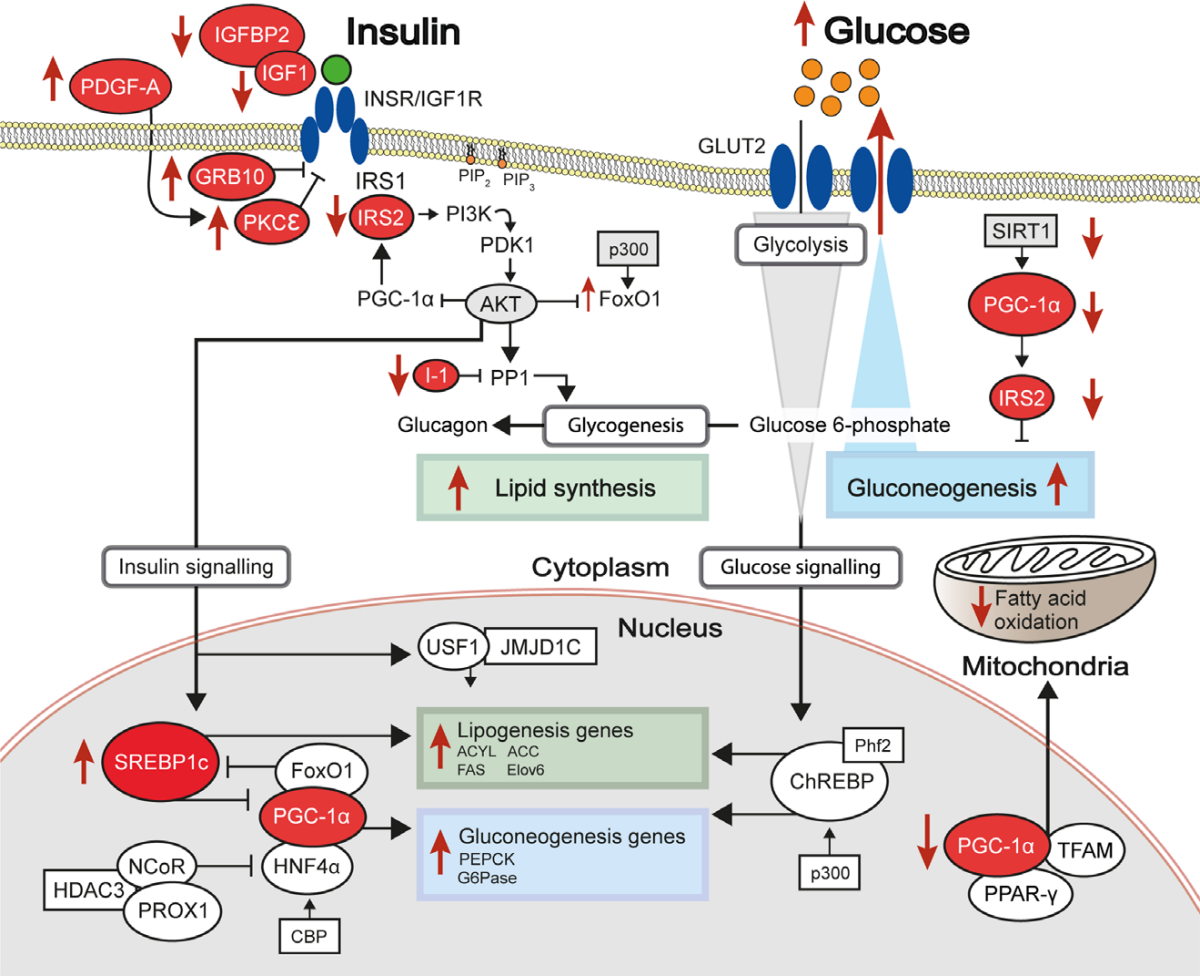
Differential methylation of insulin signalling pathway genes in insulin resistant states. The insulin signalling pathway, with key proteins encoded by genes showing differential levels of DNA methylation in insulin-resistant states highlighted in red. Red arrows indicate increased (up) or decreased (down) gene expression and activity of the indicated signalling pathways in hepatic insulin resistance.

The coordinated activity of such DNA elements, which depends on the presence of specific epigenetic marks, has important roles in both defining hepatic cell identity and activating liver-specific metabolic programs. Cellular identity is defined during embryonic development and cell lineage commitment, as well as in response to external signals. For example, the epigenome of liver-resident macrophages (Kupffer cells) is defined by signals produced by liver sinusoidal endothelial cells (23). On the other hand, epigenetic marks can be dynamically acquired and erased in response to stimuli such as nutritional intake. The epigenetic machinery (shown in **Figure 1**) interacts with master regulators of lipogenic and glycolytic gene expression programs (shown in **Figure 2**). HDACs and other histone modifiers which do not have sequence specificity *per se* can be recruited to the DNA by tissue-specific TFs such as HNF4α. This is the case in the example of HDAC3-PROX1, in which the histone deacetylase, HDAC3, interacts with the homeobox TF, prospero-related homeobox 1 protein (PROX1), with their extensive colocalization recently shown by the Lazar team to regulate a broad gene expression program that controls liver lipid homeostasis (24). Further examples include the histone acetyltransferases, CREB binding protein (CBP) and p300, which act as transcriptional coactivators of *FOXO1*, a TF that mediates lipogenic and gluconeogenic gene expression programmes (25, 26), and ChREBP, a carbohydrate responsive TF which is a master regulator of lipid and glycolytic metabolism, as well as enhanced lipogenesis in IR states (27). The histone demethylases Phf2 and JMJD1C also contribute to the activity of ChREBP and USF1, a TF which facilitates the transcription of lipogenic genes such as *FASN, ACC, ACLY* and *SREBP1C* in response to insulin or feeding (28), respectively. These examples highlight an important interaction between the availability of histone remodelers and the response to nutritional states.

## DNA Methylation Changes in the Insulin Resistant Liver

DNA methylation is the most widely studied epigenetic modification, partly due to the comparative ease of extraction and lower demand of material, compared to chromatin immunoprecipitation (ChIP) and similar methods that assess histone posttranslational modifications. This section provides an overview of observational studies which have associated hepatic IR and related diseases with altered DNA methylation, at the levels of both targeted and global association studies. Such studies have uncovered potential new aetiological leads by reporting widespread or specific changes in the epigenome and subsequent gene expression changes.

### Candidate Genes: Targeted DNA Methylation Analysis

Largely driven by prior genetic associations between specific loci and IR or IR-related diseases and by observations of differential gene expression between healthy and IR states, a number of studies has investigated the association between liver DNA methylation changes at specific loci and hepatic IR (**Table 1**). In this section, we describe key examples of candidate genes studies which explore the multi-layered nature of epigenetic regulation and the resulting coordinated changes in hepatic gene expression and insulin sensitivity.

**Table 1:** Genes investigated in targeted and candidate gene studies in liver DNA methylation studies of IR-related diseases.

Gene(s)	Direction of effect^1,2^	Associated disease/trait	Study
*DPP4*	Hypo	NAFLD (advanced *vs* mild)	(29)
*FADS2*	Hypo	NAFLD	(30)
*CYP1A1, CYP1A2, CYP2C19*	Hyper	NAFLD	(31)
*IRS2*	Hypo (intronic CpG), Hyper (promoter CpG)	T2D	(32)
*MT-ND6*	Hyper	NAFLD (advanced *vs* mild)	(33)
*PARVB, PNPLA3*	Hypo (*PARVB*), Hyper (*PNPLA3*)	NAFLD (advanced *vs* mild)	(34)
*PPARGC1A, TFAM*	Hyper	NAFLD, fasting plasma insulin, HOMA-IR	(35)
*SLC22A1, SLC22A3, SLC47A1*	Hypo	Metformin treatment in T2D patients	(36)
*TGFB1, PDGFA, PPARA, PPARG*	Hypo (*TGFB1, PDGFA*), Hyper (*PPARA, PPARG*)	NAFLD (severe fibrosis *vs* non-fibrotic liver)	(37)

^1^Hyper, hypermethylated in IR or IR-related disease.

^2^Hypo, hypomethylated in IR or IR-related disease.

#### IRS2

**Figure 3:**
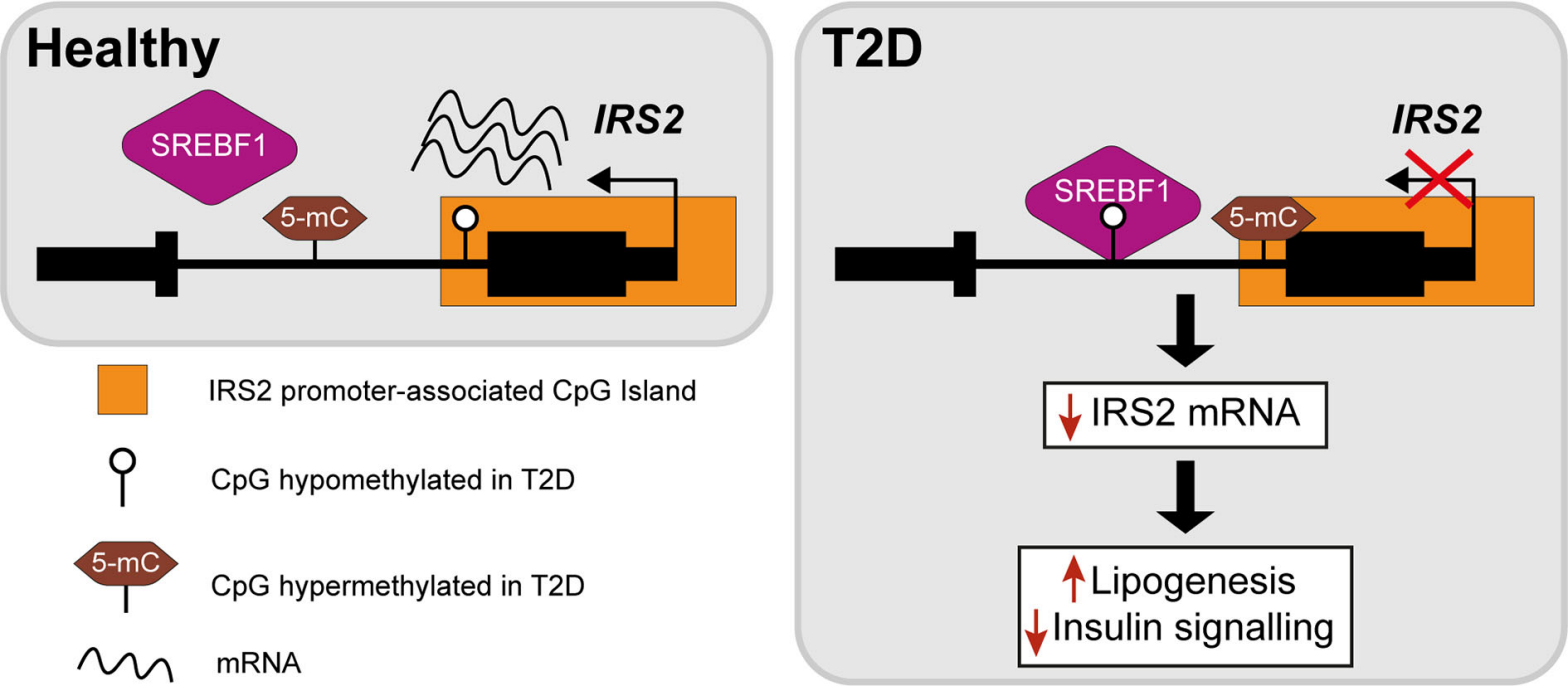
Epigenetic dysregulation of the IRS2 locus in the livers of T2D patients. Comparison of obese T2D and non-T2D revealed that the IRS2 locus, which encodes a core mediator of insulin signalling in the liver, is differentially methylated in the livers of T2D patients (41). Observed changes include the hypermethylation of a CpG site near the promoter of *IRS2* and hypomethylation of an intronic SREBF1 binding site. SREBF1 has been previously shown to interfere with the binding of transactivators to *IRS2* (42). Ultimately, these changes lead to the decreased expression of IRS2 in hepatocytes, effectively reducing insulin signalling and enhancing lipogenesis.

The insulin receptor substrates IRS1/IRS2 are scaffold proteins that are recruited to the plasma membrane when the insulin receptor becomes activated upon insulin binding (38) (**Figure 2**). In hepatocytes, IRS2 is a core mediator of insulin signalling. Mice with global ablation of *Irs2* show IR in the liver but not in skeletal muscle and present a diabetes-like phenotype (39, 40), while the decreased ratio of IRS1:IRS2 has been implicated in selective hepatic insulin resistance in T2D and NAFLD (41). *IRS2* therefore constitutes a logical candidate for targeted investigations of IR. Recently, Krause et al. investigated whether liver DNA methylation changes contributed to the dysregulation of IRS2-mediated signalling that is observed in IR-related conditions (32). The study confirmed a decrease in the expression of *IRS2* in the livers of obese individuals with T2D and identified three CpG sites in the vicinity of *IRS2* with T2D-associated methylation changes (32). These included a hypermethylated site located in the CpG island near the promoter of *IRS2*, in addition to an intronic hypomethylated CpG containing a binding site for SREBF1, which was previously shown to repress *IRS2* expression by interfering with the binding of transactivators to the *IRS2* promoter (42) (**Figure 3**). In combination, the observed hypermethylation of a promoter-proximal site, where methylation generally associates with transcriptional repression, as well as the hypomethylation of a SREBF1 binding site, suggest that coordinated changes in DNA methylation could contribute to the downregulation of this gene in T2D (**Figure 3**). Given the central role of this gene in IR, it may be of significant interest to further investigate the epigenetic regulation of *IRS2*, including in different contexts and in stratified patient groups such as individuals with T2D who show severe IR (43).

#### PPARGC1A

One of the earliest targeted DNA methylation studies of IR was conducted by Pirola and colleagues over a decade ago (35). Following the established link between IR and mitochondrial dysfunction in NAFLD (44), the authors investigated whether there was evidence of altered methylation at *PPARGC1A*, which encodes the peroxisome proliferator-activated receptor gamma coactivator 1α (PGC1α), a fasting-induced transcriptional coactivator that regulates mitochondrial biogenesis (45). PGC1α disruption contributes to hepatic IR (46, 47) and its binding is enriched at sites of NAFLD-associated changes in DNA methylation (48). PGC1α may act in part by decreasing the IRS1:IRS2 ratio, with the increased levels of IRS2 effectively amplifying the insulin-dependent suppression of gluconeogenesis (49) (**Figure 2**). The degree of hepatic *PPARGC1A* methylation was reported to correlate with the measure of insulin resistance, HOMA-IR^3^, and plasma fasting insulin across NAFLD-affected and healthy individuals, and *PPARGC1A* was markedly hypermethylated in the livers of NAFLD patients (35). Methylation of the *PPARGC1A* promoter was also inversely correlated with its expression, consistent with the role of promoter methylation in repressing gene expression (35). Interestingly, the authors also observed that mitochondrial DNA content was inversely correlated with HOMA-IR and *PPARGC1A* methylation (35). Whilst this observational study does not enable the delineation of a causal relationship between *PPARGC1A* methylation and IR, the results implicate DNA methylation as an additional mechanism in the link between hepatic IR and mitochondrial function.

#### FADS2

Hepatic lipid accumulation is highly associated with IR (50) and, for this reason, a number of candidate gene analyses have focused on hepatic lipid metabolism genes, particularly on those pinpointed by genetic association studies. A number of GWAS have implicated *FADS2* in IR-related diseases (51–53). FADS2 (delta-6 desaturase) is a rate-limiting enzyme in long-chain polyunsaturated fatty acids biosynthesis) that holds a pivotal role in lipid homeostasis. The relationship between hepatic lipids and insulin sensitivity involves a complex network of coordinated enzymatic activity, including that of the hepatic TF sterol-regulatory-element-binding protein 1c (SREBP1c) (50), a key regulator of lipogenesis and lipid homeostasis (**Figure 2**), which was disrupted in animals with FADS2 deficiency (54) (**Figure 2**). FADS2 deficiency also triggered the overexpression of other enzymes involved in fatty-acid metabolism and hepatic IR (54). Recently, an intronic *FADS2* genetic variant associated with T2D (rs174616) (55) was shown to be associated with *FADS2* methylation as well as decreased arachidonic acid/linoleic acid ratios, which is reflective of lower enzymatic activity (30). *FADS2* expression and serum FADS2 activity were also inversely correlated with the methylation of two CpG sites within a nearby enhancer and the CpG-rich region upstream of the *FADS2* transcription start site, further implicating methylation levels in the regulation of FADS2 activity (30). Due to the cross-sectional study design, the authors could not establish a direct causal relationship between DNA methylation, *FADS2* expression, and desaturase activity, however the results reveal a potentially important layer of gene expression regulation in this locus.

#### Drug Metabolism Genes

The liver is the primary site of drug metabolism and a growing body of evidence is linking defects in hepatic drug metabolism and IR. For instance, polymorphisms at loci encoding drug metabolism genes have been reported to influence insulin response (56). It has also been proposed that T2D and related inflammatory processes may alter drug pharmacokinetics and response (57). A targeted analysis of 32 genes involved in drug metabolism, comparing NAFLD patients with non-NAFLD controls, identified several drug metabolism genes with NAFLD-dependent methylation changes (31). For example, the genes *CYP1A1, CYP1A2* and *CYP2C19*, which encode enzymes that act as the initial metabolisers of drugs (phase I metabolising enzymes), were all found hypermethylated and downregulated in NAFLD (31). An additional example of the potential implications of the epigenome in drug metabolism comes from studies of patients with bipolar disorder, for whom the use of second-generation antipsychotics has been proposed to associate with IR *via* global changes in the methylome (58).

#### Fibrogenic Genes

IR is an important risk factor for hepatic fibrosis and is a proposed predictor of severe fibrosis in the context of both NAFLD and hepatitis C (59). Multiple environmental and genetic factors contribute to hepatic fibrosis and, consequently, its presentation varies considerably amongst patients. These characteristics have limited the identification of molecular mechanisms that promote the progression of chronic liver disease to fibrosis. In 2015, Zeybel et al. investigated whether the differential DNA methylation of pro- or anti-fibrogenic gene networks in liver could distinguish patients with severe fibrosis due to NAFLD or alcoholic liver disease (ALD) from those with simple steatosis (37). The authors observed that the promoters of fibrogenic genes, such as *TGFB1* and *PDGFA*, were hypomethylated in patients with severe fibrosis, compared to those from patients with mild NAFLD. In contrast, the anti-fibrogenic genes *PPARA* and *PPARD*, encoding PPARα and PPARδ respectively, were found hypermethylated in those patients (37). These observations were specifically associated with fibrosis severity, as anatomical location, age and gender did not impact the levels of methylation of those genes in control livers. The hypermethylation of anti-fibrogenic genes is particularly interesting in the context of IR, as the activation of either PPARα or PPARδ in rodents leads to improved insulin sensitivity (60, 61). Overall, this targeted study suggests that there are coordinated DNA methylation changes that promote disease worsening in patients with severe liver disease, in this case with promotion of fibrogenic gene expression and concomitant silencing of anti-fibrogenic genes, analogous to observations made in other disease settings (62).

### Genome-Wide DNA Methylation Studies

Today, it is possible to quantify DNA methylation at base-pair resolution across the genome using whole-genome bisulphite sequencing (WGBS), although DNA methylation arrays are more frequently used when comparing cohorts of cases and controls due to their lower cost. These technologies have facilitated the undertaking of epigenome-wide association studies (EWAS) of DNA methylation across multiple IR-related states. A summary of the hepatic IR-related EWAS performed to date in human liver and blood is provided in **Table 2** and key examples are discussed below, with a focus on two main pathologies that are characterised by hepatic IR: T2D and NAFLD.

**Table 2:** EWAS of IR and IR-related diseases.

Disease/trait^1^	Tissue	Cohort size	Differential methylation	Loci reported^2^	Study
HOMA-IR	Blood	332 participants	798 CpGs	*CLCA4, LECT1, CXCR1, HDAC4, IGFR1, LEPR, ABCG1, SH3RF3, MAN2C1*	(63)
T2D	Liver	60 controls, 35 cases	251 genes	*GRB10, PPP1R1A*, *IGFBP2, ABCC3, MOGAT1, PRDM16*	(64)
		11 controls, 13 obese non-diabetic, 11 obese T2D	5,682 CpGs (3,058 genes)	*PRKCE, PDGFA*	(65)
		96 controls, 96 cases	381 DMRs^3^	*PDGFA*	(66)
		23 controls, 23 cases	185 CpGs	*SYT7, LTBR, CATSPER2, LPAL2, NCALD, ZDHHC11, LGTN, OXT, PRSS21*	(67)
	Blood	10 controls, 10 obese T2D	74 DMRs	*SLC19A1, EFNA2*	(68)
		4,808 (discovery), 11,750 (replication)	28 CpGs	*LETM1, RBM20, IRS2, MAN2A2*, 1q25.3*, FCRL6, SLAMF1, APOBEC3H*, 15q26.1	(69)
		1,590 controls, 1,074 cases	5 genes	*ABCG1, PHOSPHO1, SOCS3, SREBF1, TXNIP*	(70)
		129 controls, 129 cases	2 genes	*ABCG1, PHOSPHO1*	(71)
		5,387 (discovery), 4,874 (replication)	278 CpGs	*ABCG1, PHOSPHO1, SREBF, NFATC2IP, KLHL18, FTH1P20*	(72)
		701 controls, 563 cases	18 CpGs	*ABCG1, SREBF1, TXNIP, PROC*, *SLC43A1*, *PHGDH, MAN2A2*	(73)
		42 monozygotic twin pairs	4 CpGs	Alu repeats methylation	(74)
NAFLD	Liver	18 controls, 45 cases	467 CpGs (292 genes)	*IGFBP2, IGF1, PRKCE, PGC1A, SREBF2, FOXA1, FOXA2, ZNF274*	(78)
		33 mild, 23 advanced	69,247 CpGs (2,503 genes)	*FGFR2, MAT1A, CASP1, COL1A1, COL1A2, COL4A1, COL4A2, LAMA4, LAMB1, CTGF, PDGFA, CCR7, CCL5, STAT1, TNFAIP8*	(75)
		34 controls, 35 simple steatosis, 26 NASH	1,292 CpGs (677 genes)	*PPARGC1A, DNMT1, HDAC9, ALKBH5, LDHB, COL4A1, ARL4C, SEMA3E, ITGB4*	(76)
		35 controls, 34 simple steatosis, 26 NASH	20,396 CpGs (594 genes)	*E2F1, TFAP2A NFKB1, HNF4A, HNF1A, SREBF1, TCF4, ETS1*	(77)
		35 mild, 25 advanced	610 DMRs^3^	*FGFR2, IGF1, MTHFD2, PTGFRN, ZBTB38, MGMT, FBLIM1, CYR61, NQO1*	(78)
	Blood	1496 (discovery), 1904 (replication)	22 replicated CpGs	*SLC7A11, SLC1A5, SLC43A1, PHGDH, PSORS1C1, SREBF1, ABCG1*	(79)
		731 (discovery), 719 (replication)	6 replicated CpGs	*SLC7A11, SLC43A1, SLC1A5, PHGDH, PSORS1C1, SREBF1, ANKS3*	(80)

^1^The primary outcomes reported in the study are shown.

^2^Note this is not an exhaustive list of all differentially methylated loci, but key genes of interest which are discussed in each paper.

^3^DMRs, differentially methylated regions. DMRs are discrete genomic sequences that contain a distinct methylation signature across a number of CpGs, enabling researchers to segregate one phenotypical group from another. Different computational methods can be used to identify DMRs, but in general terms, the number of CpGs used to define a DMR depends both on the type of method used to profile DNA methylation and on the distribution of CpGs at specific loci.

Few studies have directly associated genome-wide DNA methylation levels with insulin sensitivity as the primary outcome, although one such study reported that methylation at *PDGFA*, which encodes platelet-derived growth factor subunit A (66) (**Figure 2**), associated with IR measured as HOMA2-IR scores. *PDGFA* methylation was subsequently implicated in a novel mechanism of hyperinsulinemia-induced hepatic IR, as *in vitro* studies confirmed that insulin exposure reduced *PDGFA* methylation and increased its expression, while the direct exposure of cells to recombinant PDGF-AA interfered with insulin signalling by inhibiting insulin-induced AKT activation (66).

In contrast, several EWAS have assessed the levels of hepatic methylation in patients with T2D or NAFLD, and studying the methylome in these states has the potential to uncover novel mechanisms involved in hepatic IR. Firstly, the livers of T2D patients have been reported to show significant DNA hypomethylation alongside significantly lower levels of the dietary methyl-donor folate (vitamin B9) in the blood (64). Sites of hypomethylation occurred near genes previously implicated in T2D genetic risk (64) or involved in hepatic glycolysis and *de novo* lipogenesis (65), suggesting increased gene expression and pathway activity. This is exemplified by *GRB10* (64, 65), encoding the growth factor receptor-bound protein 10, which acts as an inhibitor of pathways regulating growth and metabolism, and has been previously implicated in T2D genetic risk and insulin sensitivity (81). Reflecting the broader genome-wide trends observed in T2D, *GRB10* presented lower hepatic methylation in individuals with T2D (64). Interestingly, more recent work in rodents has revealed an important axis between the activation of *Grb10* and the promotion of hepatic steatosis (82). In this study, liver-specific GRB10 ablation suppressed to a large extent the lipogenic gene programme and steatosis that are induced by acute endoplasmic reticulum stress (82). It is therefore likely that the T2D-associated DNA methylation changes affecting *GRB10* lead to its activation and hence promotion of hepatic steatosis and IR. More generally, significant changes in hepatic DNA methylation can also act as molecular fingerprints of specific metabolic states and have been used to discriminate between diabetic and non-diabetic individuals (67).

Hepatic IR is also deeply interconnected with NAFLD, since studies have shown that IR promotes the progression from simple steatosis to non-alcoholic steatohepatitis (NASH) (83), where hepatocyte injury and portal and lobular inflammation are also present, but also that hepatic steatosis and liver injury can interfere with insulin signalling (84). The EWAS carried out to date for NAFLD underscore the disruption of hepatic insulin signalling by DNA methylation as a major process underlying this disease, with many genes of this pathway showing differential methylation and associated changes in gene expression in NAFLD (some of which are highlighted in **Figure 2**). Furthermore, differential methylation in NAFLD was reported to promote inflammation, fibrogenesis, mitochondrial dysfunction and impaired lipid metabolism (75, 76, 78). Another important finding from these studies is the observation that some of the changes detected in the liver methylome reflect the progressive nature of NAFLD (75, 76, 78). In these studies, more advanced stages of NAFLD associated with global hypomethylation and concomitant over-activation of a pro-fibrogenic gene programme, as exemplified at *FGFR2* (75). Fibroblast growth factor signalling is essential in normal liver function and its dysregulation is observed in chronic liver disease, including overexpression of fibroblast growth factor receptors (FGFRs) in hepatocellular carcinoma (85). In it perhaps not entirely unexpected that the methylation of *FGFR2* was found markedly altered in advanced NAFLD, with 23 CpG sites hypomethylated, promoting its overexpression and the establishment of an inflammatory and pro-fibrotic niche (75). This trend of hypomethylation in advanced NAFLD, similar to observations in T2D (64, 65), may be of interest for the design of novel interventions to improve insulin sensitivity (addressed in more detail in the section **“Epigenetic modifications can be long-term: Dietary methyl donors”**). Interestingly, de Mello et al. reported only one site of differential methylation in cases with simple steatosis, compared with 1,292 sites in those with NASH (76), suggesting that prolonged metabolic disruption is associated with acquired changes in the DNA methylome.

One potential limitation of these studies is the association of differentially methylated sites with the closest gene, which is not always the target gene of *cis*-regulatory elements due to the 3D conformation of DNA in the nucleus (17). Nevertheless, TF binding motif analysis of NAFLD differentially methylated sites revealed strong enrichments for binding motifs of *bona fide* hepatic regulators of glucose and lipid metabolism such as PGC1α, SREBF2, FOXA1, and FOXA2 (48), further supporting the notion that appropriate DNA methylation is necessary for overall hepatic metabolic homeostasis.

Liver biopsies can provide important insight into liver-specific disease mechanisms in a research setting. However, their invasive nature limits the number of samples available and therefore the statistical power with which to detect associations. This is reflected by the fact that none of the EWAS discussed above included more than 50 cases (see **Table 2**). An alternative is to use the blood methylome as a proxy to detect changes in the liver, since cell-free DNA of liver origin can be detected in the blood, as can liver-specific DNA methylation patterns (77, 86). Blood samples can be easily obtained for large numbers of individuals, providing greater statistical power to research studies. In addition, biomarkers identified in blood are more likely to be useful in a clinical setting, where blood tests will be more appropriate for the early detection of IR. Two recent EWAS performed on whole blood used cohort sizes of 1,450 and 3,400 individuals to detect significant associations between CpG methylation and hepatic steatosis, as well as the levels of liver enzymes (79, 80). One example finding is the hypomethylation of cg08309687 (at *LINC00649*) which was associated with both NAFLD and T2D, suggesting it could be a robust biomarker of both hepatic fat accumulation and T2D (79). Other EWAS of blood samples from T2D and IR cases are listed in **Table 2** and reviewed in detail in (87). These have identified CpG sites at which differential methylation associated with measures of insulin sensitivity and future risk of T2D, including at *SREBF1* (69), which encodes the sterol regulatory element binding transcription factor 1 (SREBF1, also known as SREBP-1), a recognised regulator of insulin action in the liver (88) (**Figure 2**).

Since DNA methylation is typically a more stable modification, it is of great interest whether these acquired changes can be reversed. This important question has been addressed in studies of the liver methylome following bariatric surgery, in which NASH-associated methylation was found to be reversible, but only at specific loci (48) (discussed in more detail in the section **“Altering the epigenome: Weight loss and exercise”**).

## Histone Post-Translational Modifications in IR States

While there have been a number of studies characterising histone marks in human liver, both in primary samples and cell lines (89), there have been limited numbers of association studies which compare histone marks in disease and healthy states. Popular methods for high-throughput profiling of histone modifications include ChIP-seq (chromatin immunoprecipitation followed by sequencing) and more recent variations such as CUT&RUN (90) and CUT&Tag (91), which require smaller amounts of starting material, although these are comparatively more technically challenging than assessing DNA methylation. One genome-wide chromatin association study of note was carried out to assess differences in chromatin marks in livers with alcoholic steatohepatitis (92), although the same design has yet to be applied to IR-related liver disease. While the last five years have seen an increasing number of studies which assess chromatin marks, genome-wide studies still cover a limited number of phenotypes and tissue types.

There is evidence, however, that broad changes in histone modifications accompany IR states. Different rodent models of hepatic IR, such as models of T2D and obesity, present with global changes in the levels of different histone modifications. For instance, T2D progression has been associated with increased global levels of H3K4me1 and H3K9me2 and decreased H3K9ac and H3K23ac (93). Systematic proteomic analysis detected 15 histone marks that were differentially abundant in mice with HFD-induced obesity (94). In a similar way to the detection of differentially expressed genes, genomic regions showing differential enrichment of histone modifications between different experimental conditions or disease states can be identified using appropriate statistical tools. For example, ~5,000 regions were found to have significantly different H3K27ac enrichment in glucose-intolerant mice fed a HFD (95). This study also presents an example of how epigenomic analysis can be integrated with the analysis of 3D chromatin interactions to further identify differences in chromatin interactions between genes and associated regulatory elements, including those with altered chromatin modifications (95).

Despite the relatively few genome-wide studies of histone marks in disease states, using ChIP-seq to investigate the causal mechanisms of disease-associated genetic variants it is now a widespread approach. Mapping liver histone marks, irrespective of disease status, can help to identify the mechanisms through which non-coding genetic variants may regulate gene expression levels. For example, ChIP-seq of chromatin marks characteristic of active promoters and enhancers in liver have been used to fine-map the likely functional variants at genetic loci associated with complex disease (96, 97). ChIP has also been used to investigate epigenetic changes after manipulating cellular pathways of IR, such as in the hepatocyte-specific ablation of *Arid1a*, which induced hepatic IR in mice and reduced H3K4me3 at the promoters of its target genes (98). Likewise, ChIP-seq can identify the binding sites of IR-related TFs and hence their target genes and pathways, as in the case of the transcriptional repressor, Zfp125 (99) and the insulin receptor (100).

## The Epigenetic Machinery and Hepatic IR

The machinery that edits the epigenome, namely proteins which deposit or remove methylation, acetylation and other modifications (**Figure 1**), have themselves been implicated in perturbed liver metabolism. Studies of the epigenome, as well as gene expression and protein activity, have reported associations between the levels or activity of epigenetic modifiers and IR-related states, suggesting widespread disruption of the epigenome. The direct perturbation of histone and DNA modifiers both *in vitro* and in animal models has demonstrated their essential roles in the maintenance of insulin and glucose homeostasis in the liver. Functional studies have also implicated the epigenetic machinery in fatty acid metabolism and hepatic lipid accumulation, although the discussion below will focus on histone and DNA modifiers that have been associated with insulin sensitivity and glucose metabolism.

### Histone Deacetylases (HDACs)

Firstly, HDACs play a widely known role in influencing insulin sensitivity and hepatic lipid metabolism through the deacetylation of both histone and non-histone proteins. Changes in histone acetylation have been reported to alter the expression of glucose-mediated gene expression (101). There are 18 HDACs in humans, divided into classes I (Rpd3-like), II (Hda1-like), III (Sir2-like) and the class IV protein (HDAC11), based on sequence similarity to yeast proteins and co-factor requirement. Despite belonging to the HDAC class of proteins, many HDACs are exclusively or partially involved in the acetylation of non-histone proteins. Classes I and II contribute majorly to histone deacetylation with class I (HDAC1, 2 3 and 8) localising to the nucleus and class IIa (HDAC4, 5, 7 and 9) localising to both the nucleus and the cytoplasm (102). The seven class III HDACs, known as sirtuins based on their similarity to the yeast protein Sir2, are further subdivided and include SIRT1, 2, 6 and 7 which can be found in the nucleus.

#### Class I HDACs

Of the class I HDACs, HDAC3 activity was observed to correlate with IR in peripheral blood mononuclear cells (PBMCs) of individuals with T2D (103). Liver-specific postnatal knockout of HDAC3 in mice induced an imbalance between carbohydrate and lipid metabolism, with increased insulin sensitivity and reduced glucose production co-occurring with severe hepatic steatosis and a dramatic increase in cholesterol production and *de novo* lipogenesis (104, 105). Unlike other class I HDACs, HDAC3 activity is triggered by binding to either one of the nuclear receptor corepressors NCOR1 or NCOR2, which together with transducing β-like 1X-linked and receptor 1 (TBL1X and TBL1XR1) and the G-protein suppressor 2 (GPS2) form the core of the transcriptional repressor complex NCoR (106). Another class I HDAC, HDAC8, has been linked to the promotion of IR in NAFLD-associated hepatocellular carcinoma (HCC) (107). HDAC8 is activated by SREBP-1, an insulin-responsive TF which increases the transcription of lipogenic genes, and HDAC8 knockdown in obesity-promoted mouse models of NASH and HCC attenuated IR, reduced triglyceride levels and reduced tumour growth, potentially through widespread changes in TGFβ and mitogen-activated protein kinase/c-Jun N-terminal kinase (MAPK/JNK) signalling (107).

#### Class II HDACs

Class II HDACs can localise to both the nucleus and cytoplasm and have a lower catalytic activity towards histone acetylation compared with class I, which depends on their recruitment into a multiprotein complex with HDAC3 (102, 108). Class II HDACs have been widely associated with glucose homeostasis. In the liver, cytoplasmic class IIa HDACs can be dephosphorylated in response to glucagon; this stimulates their transport into the nucleus, whereupon they associate with the promoters of gluconeogenic enzymes, recruit HDAC3 and deacetylate their targets (109). Class II HDACs regulate several other important TFs, for example, HDAC4 catalyses the SUMOylation of the corepressor, DACH1, which can interfere with insulin signalling through the repression of *Atf6* transcription (110). Suppressing class IIa HDACs in diabetic mice and specifically in mouse liver suppressed gluconeogenesis and resulted in lower blood glucose levels (109). Since class II HDACs have a lower catalytic activity than class I, they have been suggested as a more tolerated therapeutic target for diabetes. Recent studies have suppressed multiple class IIa HDACs in tandem, however the resulting *in vitro* suppression of gluconeogenic genes was not reflected by a suppression of glucose production *in vivo* in mice (111, 112).

#### Class III HDACs

**Figure 4:**
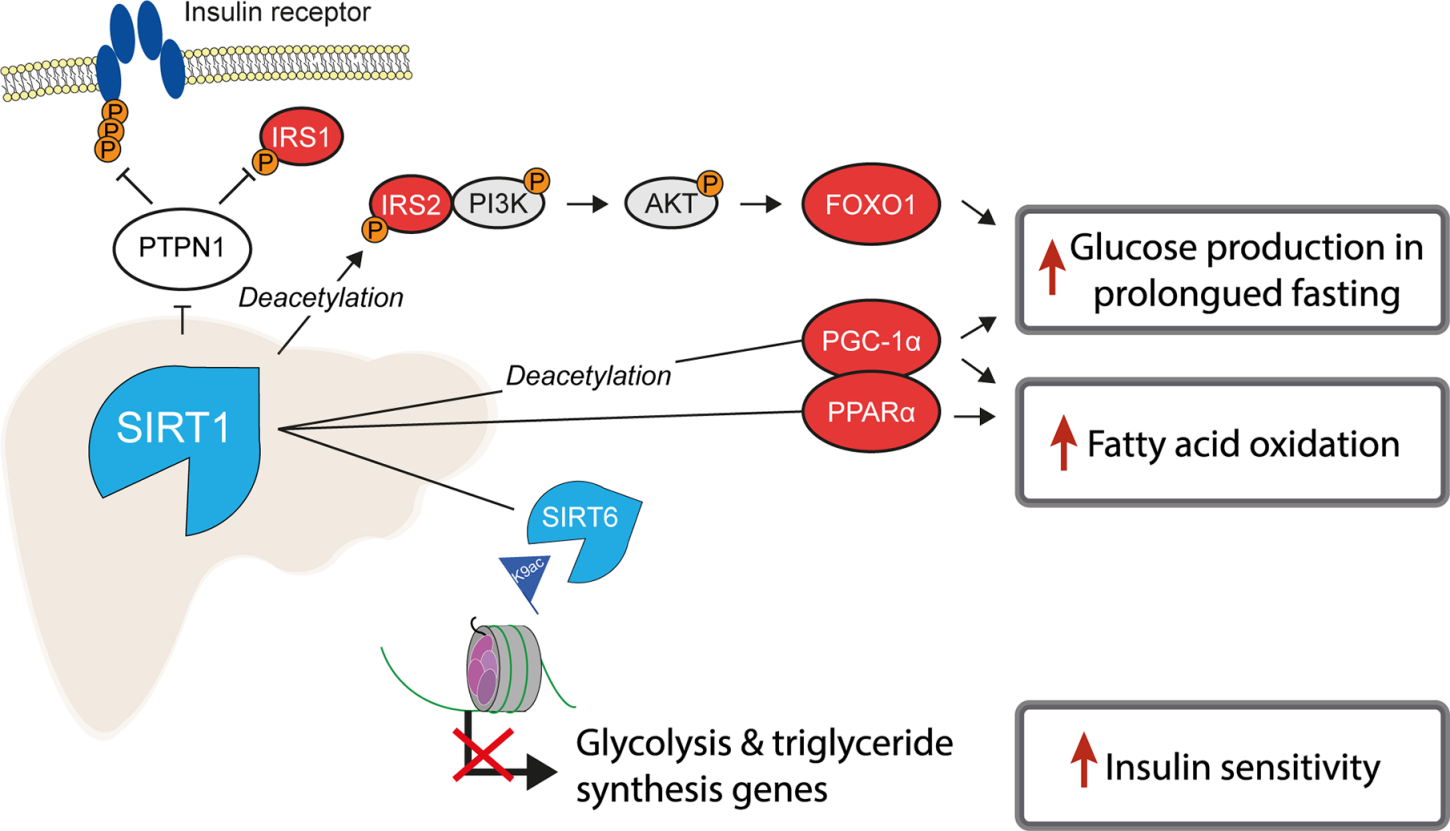
SIRT1 is a master regulator of hepatic insulin sensitivity. Under normal conditions, the functions of the histone deacetylase SIRT1 include to increase fatty acid oxidation and glucose production, by deacetylating both histone and non-histone proteins, including several key transcription factors which regulate gluconeogenic and lipogenic gene expression. Non-histone targets of SIRT1 are labelled with “*Deacetylation*”.

Of the class III, NAD^+^-dependent HDACs, SIRT1, 6 and 7 are predominantly found in the nucleus, of which SIRT1 is the major contributor to histone deacetylation. A number of studies have highlighted the role of SIRT1 as a key metabolic sensor in the liver, with some proposing it as a potential pharmacological target to ameliorate IR and T2D (113, 114). The roles of sirtuins and SIRT1 in particular are broad and diverse, not being restricted to the deacetylation of histones (115). In the liver, SIRT1 has been reported to have decreased expression in insulin resistant cell lines and tissues from HFD-fed rodents (116, 117) and its loss or inhibition led to IR in a number of studies (118, 119). SIRT1 is dramatically induced in fasting (120), leading to the deacetylation of the transcriptional co-activator PGC-1α and concomitant raise in glucose production (121) (**Figure 2**). Additionally, SIRT1 acts as a positive regulator of insulin signalling at multiple levels, both as a histone deacetylase and as a non-histone deacetylase (**Figure 4**). For example, SIRT1 represses the expression of *PTPN1*, a negative regulator of the insulin signalling cascade, improving insulin sensitivity (116), and is necessary for insulin-induced IRS-2 deacetylation (122). Moreover, liver-specific SIRT1-knockout led to disruption of mTorc2/Akt signalling downstream of the insulin receptor (119).

Another class III HDAC, SIRT6, is also tightly linked with glucose and lipid metabolism (123). SIRT6 is regulated by SIRT1 and removes H3K9ac from the promoters of genes involved in glucose and lipid metabolism (124). Liver-specific SIRT6 knockout increased hepatic insulin sensitivity in female, but not male mice (125), although was independently reported to cause fatty liver (124). Treating mouse models of T2D with a SIRT6 inhibitor improved glucose tolerance and reduced circulating levels of triglycerides, insulin and cholesterol (126). In contrast to the liver-specific effects, whole-body SIRT6 knockout in mice is lethal and induces severe hypoglycaemia (127), while whole-body overexpression was also reported to increase hepatic insulin sensitivity (128).

### Histone Acetyltransferases (HATs)

While HDACs catalyse the removal of acetylation from histones, HATs catalyse its addition. These include CBP and p300, which have been implicated in hepatic insulin sensitivity. Under physiological conditions, insulin inhibits gluconeogenesis by selectively disrupting the interaction between CBP/p300 and the cyclic AMP-responsive element-binding protein (CREB), a TF involved in the expression of metabolic and gluconeogenic genes (129). In functional studies, p300 overexpression in mice was reported to cause hepatic steatosis, insulin resistance, and inflammation (27). Inhibiting CBP/p300 in mouse liver decreased hepatic lipid content and the expression of lipogenic genes *in vivo* and also reduced the expression of gluconeogenic genes in primary mouse hepatocytes (130). While this study reported no difference in *in vivo* insulin sensitivity, mice had reduced fasting glucose and lower glucose levels after glucose loading (130). Similarly, CREB activity is positively associated with hepatic IR and NAFLD in mice (131) and its knockdown in mouse liver decreased hepatic and circulating lipid levels and improved insulin sensitivity (132). The p300/CBP‐associating factor (PCAF) is another HAT that acetylates histone H3 and other non-histone proteins including key regulators of gluconeogenesis (PGC-1α) (133) and lipogenesis (ACLY), effectively regulating insulin sensitivity and glucose production in the liver (134), while p300 is also known to acetylate and activate FOXO1 (109) (**Figure 2**).

### Histone and DNA Methyltransferases (HMTs/DNMTs) and Histone Demethylases (HDMs)

In comparison to the more widely studied histone acetylation, there have been fewer studies linking HMTs, HDMs and DNMTs to hepatic IR. Firstly, the major family of HMTs is the SET domain family, consisting of over 55 proteins in humans, of which around half have no known substrates and may have no enzymatic activity (135, 136). As an example of an association between a HMT and hepatic IR, haploinsufficiency of the HMT *MII2* resulted in hyperglycaemia and hyperinsulinemia at fasting and peripheral IR, as well as hepatic fat accumulation and abnormal plasma triglycerides in mice (137). Another HMT, G9a, which catalyses the addition of the repressive histone marks, H3K9me1 and H3K9me2, was observed to have lower expression levels in diabetic mouse models (138). However, G9a has been reported to impact insulin signalling through a methyltransferase-independent mechanisms, by regulating the architectural TF HMGA1, a key regulator of the insulin receptor (138). There are other noteworthy interactions between HDMs and TFs; for example, the HDM Phf2 is required for transcriptional activation by the glucose-responsive TF ChREBP, by erasing the repressive mark H3K9me2 (139) (**Figure 2**). ChREBP is a master regulator of lipid and glycolytic metabolism and plays a major role in enhancing lipogenesis in insulin resistant states (140). Liver-specific knockout of the HDM JMJD1C, which interacts with USF1 to promote the transcription of lipogenic genes, resulted in reduced expression of lipogenic genes and reduced lipogenesis (28).

In relation to DNA methylation, the mammalian DNA methyltransferases include DNMT1, DNMT3a and DNMT3b (141). DNMT3a has a well-known role in peripheral IR in adipose tissue (142). In the liver, DNMT1 regulated the expression of miR-9-3 in mice, which has been found to regulate hepatic glucose production and insulin sensitivity (143). Knockout of TET1, which catalyses the first step of DNA demethylation (the conversion of 5-methylcytosine into 5-hydroxymethylcytosine), inhibited NAFLD progression in mice and promoted fatty acid oxidation by activating PPARα through increased hydroxymethylation at the *PPARA* promoter (144).

## Altering the Liver Epigenome

**Figure 5:**
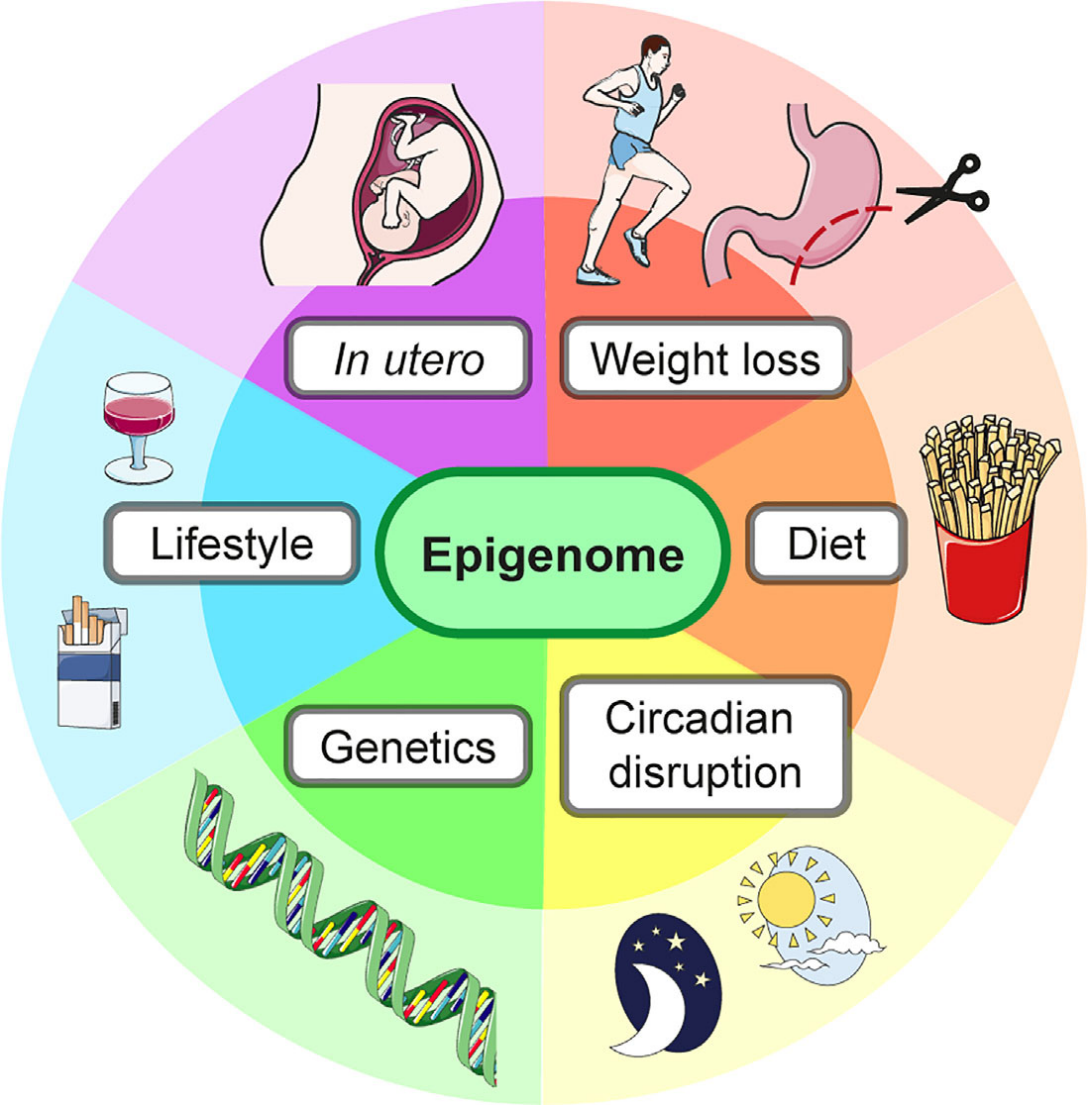
The liver epigenome is influenced by many factors. The epigenome and therefore gene expression, including of metabolic gene programs, can be altered by external factors as well as the underlying genetic sequence.

Changes to the liver epigenome can result from multiple factors, examples of which are shown in **Figure 5**. The epigenome has transient, flexible changes which cycle with changes in gene expression, including in response to dietary intake and the time of day. On the other hand, long-term epigenetic marks can define cell-specific gene expression programs. Both transient and long-term changes to the epigenome in the context of hepatic IR are discussed below.

### Epigenetic Modifications Can Be Long-Term

#### Parental, *In Utero* and Postnatal Exposures

Following the observation that the epigenetic machinery may contribute directly to hepatic IR, as well as the presence of various IR-associated epigenetic marks, a question that remains is whether deleterious epigenetic marks are persistent and to what degree, since long-term modifications may themselves become causes of disease as manifestations of earlier exposures. This question has been addressed by studying how specific early-life exposures can increase the long-term risk of metabolic disease. For example, both maternal overnutrition and famine during pregnancy have been linked to an increased risk of IR and cardiometabolic disease in offspring (145), as have a low birth weight (146), exposure to gestational diabetes (147) and childhood overnutrition (148). The epigenome may be one factor driving, or at least reflecting, this long-term risk. For instance, the levels of DNA methylation in neonatal blood spot samples have been reported to associate with IR in children at five years of age (149).

The specific impacts of parental exposures on the liver epigenome of offspring have been studied using animal models. Both paternal and maternal metabolic syndrome can lead to epigenetic reprogramming and IR in offspring. A recent study by De Jesus et al. demonstrated that wild-type mice with one LIRKO (liver-specific insulin receptor knockout) parent, either maternal or paternal, were characterised by hepatic and whole-body IR (150). The offspring liver showed altered gene expression including for pathways of cholesterol, triglyceride fatty acyl-CoA synthesis and AKT signalling (**Figure 2**) and widespread changes in the DNA methylation, particularly at the promoters of genes involved in cholesterol synthesis, MAPK, AKT, insulin and TGF-β signalling (150).

Another widely studied model is that of maternal HFD. Offspring typically show elevated blood glucose levels and IR along with epigenetic modifications in the liver, including widespread hypermethylation and altered gene expression along with changes in the enrichment of active promoter histone marks (e.g. H3K14ac and H3K9me3) in mice and non-human primate offspring (151–153). These changes are particularly observed at genes involved in both metabolism and liver development (153, 154). Examples of specific alterations include reduced repressive and increased active histone marks at the *Pck1* gene, which encodes a rate-limiting enzyme in gluconeogenesis (PEPCK), and hypermethylation of the *Irs2* gene (**Figure 2**) (155). Maternal HFD also led to the altered expression of genes encoding the epigenetic machinery in offspring, particularly those in the histone acetylation pathway such as SIRT1 (152, 153). Notably, SIRT1 overexpression has been reported to attenuate the effects of a maternal HFD in offspring and protect against hepatic steatosis and insulin resistance (156). It is also of interest that the metabolic alterations seen in offspring persist despite a normal diet (155), however others have observed a ‘latent metabolic phenotype’ in which IR developed in the offspring of obese mice only when exposed to a Western-style diet (154). This difference may be attributed to the type and timing of early-life exposure. In a model of neonatal overfeeding in mice, monoacylglycerol O-acyltransferase (*Mogat1*) was identified as a potential early mediator of hepatic IR and steatosis, with a 3-fold upregulation accompanied by a 50% reduction in the enrichment of the repressive histone mark H3K27me3 (157). The Mogat1 enzyme catalyses the conversion of monoacylglycerol to diacylglycerol (DAG), a molecule which may directly interfere with the insulin signalling cascade (158).

#### Dietary Methyl Donors (*In Utero*)

Hepatic insulin sensitivity can also be influenced by the intake of specific dietary components which contribute to epigenomic maintenance. In mammals, the universal methyl donor S-Adenosyl methionine (SAM) provides methyl groups for the methylation of DNA, RNA and histones, and the levels of DNA and histone methylation depend on the availability of SAM (159). SAM synthesis is influenced by the dietary intake of its precursor, methionine, as well as co-factors folate (vitamin B_9_), betaine and vitamins B_2,_ B_6_ and B_12_ (160). Serum levels of these methyl-donor metabolites, including folate and B_12_, have been associated with IR (161, 162). The effects of *in utero* exposure to folate are of particular interest, since folate supplements are recommended for pregnant women to reduce the prevalence of neural tube defects and other congenital malformations (163). Despite these benefits, *in utero* exposure to high levels of folate in the later stages of pregnancy^4^ has been associated with adverse immune and metabolic outcomes in offspring (163), including an increased risk of developing IR during childhood which may be exacerbated by vitamin B_12_ deficiency (164, 165).

In terms of the epigenetic impact, dietary levels of folate can influence the methylome (166, 167) and DNA methylation changes in offspring exposed to varying folate levels *in utero* have been reported to persist into adulthood (168). Studies of the epigenome in human infants are largely limited to accessible tissues such as cord blood, plasma and buccal cells, in which folate levels during pregnancy have been associated with distinct methylation profiles (169, 170). Alternatively, animal models have been used to investigate the effects of maternal folate levels on the liver epigenome. Consistent with observations in humans, excess maternal folate has been associated with IR in rodent offspring (171, 172), which is accompanied by aberrant DNA methylation of important lipid metabolism genes such as *ATGL*, which encodes for a major hepatic lipase (171), and increased hepatic lipid synthesis (173). Maternal folate supplements have been associated with decreased levels of global DNA methylation in offspring liver (174). This negative relationship is surprising, although similar results have been reported elsewhere (175) and has been suggested to result from the altered methylation of DNMTs (174, 175). It is also worth noting that folate has other functions aside from acting as a methyl donor, for example regulating the lysine demethylase LDS1, such that folate deficiency leads to increased levels of active histone marks H3K4me1/2 in the liver of rodents (176). Finally, an interesting observation is that the metabolic phenotype of mice exposed to excessive folate *in utero* may be modulated by their dietary composition in later life. Feeding a HFD to mouse offspring exposed to high levels of folate *in utero* was reported to induce IR and glucose intolerance (177), while a high-folate diet improved glucose tolerance (172). These results hint at a potential role of *in utero* folate for priming metabolic pathways.

#### Dietary Methyl Donors (Adulthood)

There has also been widespread study into the effects of dietary supplements during later life. It is well established that serum levels of folate are lower in individuals with IR (161, 162). In animal models, folate supplements have been associated with increased hepatic lipid metabolism and reduced steatosis in response to HFD (178), while chronic folate deficiency caused hepatic IR and lipid accumulation (179). In humans, several meta-analyses of randomized control-trials have reported that folate supplements either had no effect on lipid levels in individuals with metabolic disease (180) or improved insulin sensitivity (180, 181). It is of interest that mice who received a high-folate diet following exposure to high levels of folate *in utero* experienced improved glucose tolerance (172); whether this effect extend to humans, such that folate supplements may benefit individuals who were exposed to high levels of folate *in utero*, will be an interesting area of future study. A second important dietary nutrient is methionine, the precursor of SAM. With respect to IR, the levels of methionine transmethylation were lower in insulin resistant patients with hepatic steatosis (182), while methionine restriction has consistently been shown to increase hepatic insulin sensitivity and protect against diabetes and metabolic dysfunction in rodents (183–185). To the extreme, however, methionine deficiency may cause liver damage (186), while methionine supplementation can attenuate oxidative stress and reduce liver damage in cases of liver disease (186, 187), in part by acting as a target for reactive oxygen species (188). Methionine may also have tissue-specific effects on the epigenome, further adding to its complexity (189).

In short, the effects of dietary methyl donors on the hepatic epigenome and IR are diverse and complex, particularly for methionine, which may discourage the use of supplements in treating IR. While there is some evidence from human studies that folate supplements may improve insulin sensitivity, there is also evidence to suggest that both deficiency and excess folate could be deleterious, with a recent study showing that both led to skeletal muscle IR in mice (190); it will be of interest to see if these observations extend to the liver. Thus, it may be premature to recommend dietary supplements to treat hepatic IR and further studies of the liver-specific mechanisms underlying their effects are required. If any such treatment were to be used, there is likely to be an optimal dietary intake which may vary between individuals.

While this section discussed the examples of dietary folate and methionine, other examples include berberine, which reduced methylation of the *MTTP* promoter and attenuated fatty liver in rats fed a HFD (191), and the prebiotic inulin, which improved glucose tolerance and differential DNA methylation in the presence of a HFD, including at the *PI3K* gene encoding a phosphoinositide kinase active in the insulin signalling pathway (192).

### Epigenetic Modifications Can Be Short-Term: Lifestyle and Environmental Exposures

As discussed above, long-term epigenetic modifications can result from external exposures, including those during development and early life. However, epigenetic modifications can also be transient or short-term, such as those obtained in response to the diet. There is also growing evidence to suggests that some long-term marks can eventually be reversed. Various lifestyle and environmental factors have been shown to influence the epigenome throughout life, either conferring deleterious or beneficial effects to insulin response. Two examples of epigenomic modifiers, the circadian rhythm and exercise, are discussed below.

#### Circadian Regulation of Insulin Sensitivity

An example of transient epigenetic gene regulation can be observed in response to the circadian rhythm. The liver circadian clock is synchronized to the hypothalamic suprachiasmatic nucleus (SCN), but can also be regulated autonomously by food intake (193). Global insulin sensitivity is known to fluctuate throughout the day following the circadian cycle, to which diurnal hepatic glucose production is a major contributing factor (194). In humans, metabolic disease and IR are associated with perturbations in the circadian rhythm (195); a classic example is the association between night shift work and an increased risk of IR and diabetes (196). Night shift workers show altered levels of DNA methylation in blood which increases with the frequency and duration of shift work completed (197, 198), suggesting that long-term exposures may reinforce epigenetic changes and disease risk.

The epigenome plays a crucial role in circadian gene expression programs; the core circadian protein, CLOCK, acts as a histone acetyltransferase (199) and epigenetic modifiers including HDAC3 bind to DNA in a circadian manner (200, 201), particularly at lipid metabolism genes where the levels of histone acetylation inversely correlate with RNA polymerase II binding and gene expression (201). The circadian TF BMAL1 stimulates the cyclic expression of glucose and lipid metabolism genes (202) and has been reported to interact with SIRT1 and p300 together in a complex with CLOCK (203, 204). Circadian cycles have also been observed for the deposition of histone acetylation and methylation marks (203–206), as well as DNA cytosine modifications (5-methyl- and 5-hydroxymethylcytosines) (207). The role of the hepatic epigenome in the relationship between circadian disruption and IR has been specifically investigated in rodent models. For example, the liver-specific knockout of the histone deacetylase SIRT1 dysregulated the hepatic circadian cycle, causing hyperglycaemia and IR (119, 208). Disrupting key circadian proteins also alters insulin and glucose homeostasis, with the liver-specific knockout of *Bmal1* causing overt hypoglycaemia and disrupting the rhythmicity of fasting blood glucose and related gene expression (209, 210), whereas liver-specific knockout of the circadian genes *Ddb1* and *Cry1* led to enhanced gluconeogenesis (211, 212).

#### Weight Loss and Exercise

As two modifiable lifestyle factors, the impact of exercise and weight loss on the epigenome and IR are of great interest. Obesity is a major risk factor for IR (213) and is associated with widespread changes in the liver epigenome (214). Childhood obesity is associated with cardiometabolic disease later in life (215) and the prevalence of obesity is significantly higher in individuals with diabetes and NAFLD compared to the general population (3). Both weight loss and increased exercise have been reported to lower the risk of metabolic disease, which seems to be at least in part mediated by epigenetic remodelling.

Several human studies have examined the liver methylome in the context of extreme weight loss following bariatric surgery, which can improve insulin sensitivity and is an effective strategy for resolving and lowering the future risk of T2D in obese individuals (216). These studies have reported that bariatric surgery may partially reverse NAFLD-associated DNA methylation in the liver (48). However, independent work suggests that bariatric surgery may be unable to reverse obesity-induced epigenetic ageing of the liver (214). A recent meta-analysis on the effects of bariatric surgery across tissues reported inconsistencies between studies, but concluded that surgery appears to reverse DNA methylation at specific loci, with consistent improvements in metabolic profiles (217, 218). This suggests that specific sites of the epigenome host more persistent modifications, the implications of which may emphasize the need for preventative intervention before persistent epigenetic marks can be deposited.

A similar conclusion may be drawn from animal studies. In mice, HFD-induced changes in DNA methylation, chromatin modifications and accessibility were to some extent reversible following the return to a normal diet and body weight, including for H3K27ac and DNA methylation (219, 220). This included restoration of high H3K27ac enrichment at regulatory elements containing binding sites of important hepatic TF families including HNF4, SREBP and C/EBP (220). However, some hepatic DNA methylation marks induced by a high-fat, high-sucrose diet were reported to be more persistent and to revert more slowly than weight loss (219). The persistence of HFD-induced epigenetic changes may depend on the genetic background (221), as well as on the tissue type. For instance, HFD-induced changes in mouse liver gene expression reverted more quickly compared to adipose tissue, while in humans, hepatic IR improved following bariatric surgery to a greater extent than adipose inflammation (222). These observations could underscore the rewriting of the liver epigenome as a potential means of improving insulin sensitivity, but it must be noted that the restoration of insulin sensitivity after a HFD may require a prolonged exposure to a normal diet due to the presence of persistent epigenetic marks (222).

Exercise is also associated with a lower risk of metabolic disease and improved insulin sensitivity in humans and animal models (223). Physical exercise is significantly associated with liver ‘fitness’, including an association between physical inactivity and NAFLD (223). During pregnancy, exercise may manifest improved glucose tolerance in older offspring (224) and maternal exercise is associated with DNA methylation changes in human cord blood (225). Human studies relating to exercise and the epigenome are largely limited to studies of blood and peripheral tissues including adipose and skeletal muscle (226), however liver-specific studies in mice have demonstrated that physical exercise may prevent hypermethylation induced by HFD and partially attenuate hypomethylation (227). The studies described here together present a scenario where lifestyle interventions such as weight loss and exercise can successfully reverse many epigenetic changes associated with metabolic disease, although prolonged interventions may be required to reverse more persistent epigenetic marks.

## Future Perspectives

### Epitranscriptomics

The emerging field of epitranscriptomics has revealed that RNA modifications can also modulate gene expression levels (228). Whilst there are over 100 types of RNA chemical modifications, N^6^-methyladenosine position (m^6^A) is the most abundant internal mRNA modification in eukaryotic cells and it plays an important role in gene expression, including through altering protein binding, mRNA structure, stability, splicing and translation efficiency (229). mRNA m^6^A methylation has recently been implicated in liver disease. For example, the levels of m^6^A methylation and the m^6^A methyltransferase METTL3 were observed to be higher in the livers of patients with T2D and positively correlated with HOMA-IR (230). Several recent studies have also reported increased hepatic m^6^A levels in mice under HFD, while knocking-out METTL3 improved insulin sensitivity and reduced lipid accumulation, including by increasing specific mRNAs half-lives and reducing the total levels of mRNA encoding the fatty acid synthase, FASN (229–231). The topic of liver disease epitranscriptomics had been extensively reviewed by Zhao et al. (232).

It is clear that the contribution of aberrant RNA modifications to IR is currently less characterised than other epigenomic features. Still, these studies highlight the importance of this epigenetic mechanism as an additional layer of gene regulation that contributes to hepatic insulin homeostasis. Some interesting insights have already been gained regarding the relationship of m^6^A levels and disruption of the circadian rhythm. For example, mice with knockout of the circadian factor *Bmal1* presented increased levels of hepatic m^6^A, leading to increased *Ppara* mRNA lifetime and expression and hepatic steatosis (233). In future studies, it will be interesting to investigate the interplay of RNA modifications and their associated epigenetic machinery with other epigenomic and genetic factors that influence insulin response.

### Treatment Opportunities

Targeting the epigenome offers the opportunity, in theory, to reverse deleterious epigenetic marks and alter gene expression levels. Multiple drugs and compounds which target the epigenome have been proposed for the treatment of cardiometabolic disease. These include HDAC inhibitors and SIRT1 activators to treat IR and T2D (234, 235). Both *in vitro* and *in vivo* studies have aimed to assess whole-body and tissue-specific effects of these epigenetic drugs. For example, HDAC inhibitors and SIRT1 activators have been reported to improve insulin secretion by pancreatic β‐cells (87, 236).

HDAC inhibitors are currently in use to treat different diseases, including cancer, thalassemia and sickle cell anaemia (102, 237), although non-selective, ‘pan’-HDAC inhibitors such as vorinostat and romidepsin have been associated with severe side effects due to the diverse biological functions of HDACs (238). Similarly, the knockdown of multiple class II HDACs caused severe adverse effects in mice and, even though it lowered the expression of gluconeogenic genes, it did not have a significant impact on gluconeogenesis (112). More selective HDAC inhibitors will likely be more appropriate for the treatment of metabolic disorders, although this is challenged by the risk of off-target effects due to the high degree of HDAC similarity. It has been suggested that HDAC8 may be an appropriate target, since it has less structural similarity with other HDAC enzymes (239). The HDAC inhibitor tannic acid was recently reported to attenuate the accumulation of lipids in the liver and prevent NAFLD in mice fed a HFD, by decreasing H3K9 and H3K36 acetylation and transcription of lipogenesis-related genes, including fatty acid synthase (*FASN*)*, SREBP1C* and ATP citrate lyase (*ACLY*) (240).

Also of interest is the effect that drugs commonly used to treat cardiometabolic disease have on the epigenome. For example, metformin, a widely used T2D treatment, has been reported to increase SIRT1 activity and decrease class II HDAC and HMT activity (241). In primary human hepatocytes, metformin treatment induced changes in chromatin modifications at an enhancer element within the *ATM* gene, a locus that has been associated with response to metformin (242), and it reversed H3K36me2 signatures in mouse models of prediabetes and diet-induced obesity (94). The protein encoded by *ATM* has a role in AMPK activation, a pathway which stimulates fatty acid oxidation and decreases glucose production and lipogenesis in liver (243). In mouse studies, metformin reversed histone marks associated with diet-induced-obesity (94) and activated AMPK, resulting in the phosphorylation of class II HDACs and nuclear exclusion of HDAC4, HDAC5 and HDAC7 in the liver (109).

In conclusion, targeting the epigenome offers the possibility to reverse deleterious epigenetic marks and reprogram unfavourable metabolic pathways. There are several therapies in use which alter the epigenome, including SIRT1 activators such as metformin and resveratrol. However, as this review has discussed, there is still much to learn about the effects that potential interventions such as HDAC inhibitors or dietary supplements may have on the hepatic epigenome and wider cellular functions. Exercise and weight loss are attractive treatment options which may effectively alter epigenomic programming and the collective evidence suggests that these interventions will be most effective if maintained for extended periods of time. Future research into treatments for hepatic IR have two main avenues (1): preventing the deposition of persistent epigenetic marks, such as those seen in NAFLD progression, and (2) developing treatments to reverse deleterious epigenetic marks and metabolic profiles.

### Epigenetic Biomarkers

Aside from potential treatments, the epigenome may also be a source of disease biomarkers. These may enable the diagnosis of disease progression without requiring invasive biopsies or may contribute towards estimates of future disease risk. For the former, novel biomarkers which can discern mild from severe disease states may prove particularly useful in a clinical setting. While IR can be effectively diagnosed using a blood test, complex IR-related states such as NASH, which is a major risk factor for cirrhosis and HCC, are difficult to distinguish from simple steatosis without a liver biopsy (244). Bloodborne biomarkers offer a potential non-invasive diagnostic. For example, circulating levels of cytokeratin 18, which is seen following hepatocyte death, has been reported as a strong candidate for distinguishing NASH patients (245). In terms of the epigenome, circulating liver-specific miRNAs may also provide rapid and non-invasive biomarkers of liver damage (246) and IR/NAFLD (247). Methylation of the *PPARG* gene in plasma DNA was reported to reflect the molecular pathology associated with fibrotic liver disease (248). Several EWAS have reported associations between methylation in blood samples and IR-related disease (see previous EWAS section). Promising studies have also identified sites of methylation which associate with the future risk of T2D onset (72).

A study by Dijk et al. analysed neonatal blood spot samples and identified 63 genomic regions which associated with insulin sensitivity at five years of age (149). Such regions could offer biomarkers which may inform appropriate lifestyle interventions to reduce risk of IR-related disease. More recently, Sadeh et al. developed a method to identify liver-specific histone modification signatures in plasma cell-free nucleosomes using ChIP-seq (249). Despite the relatively small sample size and patient heterogeneity, the authors were able to detect liver-disease associated changes in histone modification enrichment at specific loci (249). This methodology is expected to be taken up in the future to facilitate the high-throughput interrogation of disease signatures in patient blood samples.

A popular area of recent discussion is in predicting disease onset using genetic risk scores (250). This approach currently has limited use in a clinical setting, although future research is likely to benefit from incorporating genetic risk scores with epigenetic marks, in order to capture acquired as well as inherited risk. It should also be noted that genetic variation can cause epigenetic variation, which should be considered in efforts to integrate genetic and epigenetic risk. Another area of consideration is the use of biomarkers in identifying subgroups of patients. For example, genetic data can be used to distinguish T2D cases characterised by severe IR, which is closely linked with the risk of complications and choice of treatments (251, 252). There is some evidence to suggest that blood methylation may predict future risk of complications in diabetic cases (253). At present, investigating epigenetic markers of IR offers immediate promise in a research setting, as they continue to uncover novel mechanisms contributing to IR. Incorporating epigenetic biomarkers in mainstream healthcare must first provide enough benefit to outweigh the costs of additional infrastructure and training.

### Open Questions and Future Directions

The studies discussed in this review support the notion that hepatic IR and its associated traits and comorbidities are not only characterised by broad changes of the liver epigenome, but also that the epigenome should be regarded as a modifier of metabolic programs and whose disruption may impact insulin sensitivity. The current studies also highlight potentially modifiable pathways to improve hepatic insulin sensitivity, but a tremendous amount of research work will be required to investigate the additional aspects of hepatic IR and the safety of so-called epigenetic drugs.

While the observational nature of many human studies still precludes the characterisation of mechanisms of disease progression at large, mounting evidence suggests that the epigenome should be regarded as a rich pool for the discovery of disease biomarkers. The observation that individuals with IR showed higher global methylation than their normoglycemic monozygotic twins emphasises the role of environmental factors in modulating the epigenome; information which can provide biomarkers independent from inherited genetic risk factors (74). This notion has truly taken off in regard to the profiling of liver methylomes in IR, as demonstrated by the large array of methylome-wide studies we discussed. The high degrees of correlation between DNA methylation patterns in blood and liver (77) make blood methylation biomarkers an appealing strategy for the early identification of individuals at risk of developing hepatic IR and related cardiometabolic conditions (254). It must be noted, however, that early DNA methylation array versions largely excluded informative methylation in the non-coding genome, instead focusing on gene promoters and bodies. Given the large body of evidence demonstrating that the non-coding genome is highly functional and hosts a myriad of different types of DNA regulatory elements (255), previous studies were inevitably limited in scope. For this reason, there has been an increased uptake by the community of true genome-wide profiling of DNA methylation landscapes with the application of techniques such as Methylated DNA Immunoprecipitation followed by sequencing (MeDIP-seq) (256), whole-genome bisulphite sequencing (WGBS) and, more recently, with the repurposing of long-read sequencing technologies to detect methylated DNA (257, 258). Whilst these techniques offer the opportunity to obtain a very detailed view of the DNA methylation profiles of different tissues and metabolic states [even of individual cells with their adaptation to single-cell use, as already demonstrated in mouse liver (259)], their application on a population level is not expected to be rolled out soon given the high cost per sample and need for training and infrastructure. Still, we do expect to obtain increased resolution in the picture of IR methylomes in the near future with the use of more recently developed DNA methylation array designs, which incorporate a higher number of distal regulatory elements. For example, the Infinium MethylationEPIC Chip [Illumina (260)], includes ENCODE open chromatin sites and enhancers. These studies will enable the increased integration of genetic associations from GWAS, which most often detect noncoding risk variants in distal regulatory elements, with EWAS signals.

Due to higher associated costs and technical challenges, the profiling of histone modifications has not really matched the pace of methylome-wide studies. Nevertheless, exciting research has recently demonstrated the potential to once more harness the correlation between liver and blood epigenomic profiles, this time by performing ChIP-seq on circulating cell-free DNA (249). Future studies are expected to leverage on this new technology to identify additional biomarkers of hepatic IR, assisting in the prevention of NAFLD, T2D and other cardiometabolic diseases, which together have an enormous health, societal and economic impact. It is also expected that future studies will provide much needed granularity to our view of liver IR epigenomes by, for instance, mapping accessible (and thus *active*) chromatin sites with single-cell resolution in liver biopsies from patients presenting varying degrees of disease severity. Importantly, recent technological advances now make it possible to perform multiomic analyses with single-cell resolution (261). Such powerful studies may revolutionise our understanding of hepatic IR and potentially uncover disease processes that are particular to one cell type, or even one sub-population of resident liver cells.

Understanding the interplay between the liver epigenome extrinsic factors such as *in utero* exposures or diet will inevitably still rely on appropriate animal models. These models are expected to remain central for studies of long-term effects on the epigenome. Still, improvements in stem cell differentiation protocols to produce mature human hepatocytes are expected to enable the further development of novel human *in vitro* models of IR. Together with rodent models, sophisticated *in vitro* models such as organ-on-a-chip will also enable the better understanding of how cues from other tissues important for glucose homeostasis, such as pancreatic islets (262), influence the liver epigenome to promote IR.

Finally, future studies of the epigenetic basis of hepatic IR should aim to better integrate epigenomic profiles with genetic susceptibility factors. This integration of genomic and epigenomic information will be further enriched by the analysis of more ethnically diverse panels, as the vast majority of current studies are centred around the analysis of Caucasian genetic risk factors. Such results are unlikely to arise from individual research labs and are instead expected to be produced by large international consortiums. Thus, a difficulty will not only be data gathering, but also standardization of data processing and homogenization. Overall, while there will be challenges ahead, the field of epigenomics is expected to offer important contributions to the identification of targetable pathways to improve hepatic insulin sensitivity.

## Author Contributions

HM, CS-C and IC wrote this review and prepared the accompanying figures. All authors contributed to the article and approved the submitted version.

## Funding

The Cebola lab was supported by the Accelerating Medicines Partnership Type 2 Diabetes program (https://fnih.org/our-programs/AMP/accelerating-medicines-partnership-type-2-diabetes-project, https://t2d.hugeamp.org/ and https://www.niddk.nih.gov/) (AMP T2D RFP16). I.C. has been awarded an Academy of Medical Sciences Springboard Fellowship (SBF005\1050), which is supported by the British Heart Foundation, Diabetes UK, the Global Challenges Research Fund, the Government Department for Business, Energy and Industrial Strategy and the Wellcome Trust. HM was supported by the High Cost Training Fund from the Imperial College MRC Supplement Scheme.

## Conflict of Interest

The authors declare that the research was conducted in the absence of any commercial or financial relationships that could be construed as a potential conflict of interest.

## Footnotes

1 Metabolic zonation refers to the distinct patterns of cellular activity along the axis from the periportal to central vein. This topic is reviewed in detail in (15).

2 In opposition to *Epigenetics* that refers to local epigenetic modifications and regulatory processes, *Epigenomics* refers to the study of collective epigenetic changes across the whole genome in a cell, entire tissue or organism.

3 Elevated HOMA2-IR (updated Homeostasis Model Assessment of Insulin Resistance) can be calculated using measures of fasting glucose and fasting insulin in the blood and is indicative of increased levels of IR in peripheral tissues, being commonly observed in patients with pre-diabetes and T2D.

4 Folate prevents neural tube defects when taken during the *early* stages of pregnancy (163).
